# Comparison and Validation of Putative Pathogenicity-Related Genes Identified by T-DNA Insertional Mutagenesis and Microarray Expression Profiling in* Magnaporthe oryzae*

**DOI:** 10.1155/2017/7198614

**Published:** 2017-02-14

**Authors:** Ying Wang, Ying Wáng, Qi Tan, Ying Nv Gao, Yan Li, Da Peng Bao

**Affiliations:** National Engineering Research Centre of Edible Fungi, Key Laboratory of Edible Fungi Resources and Utilization (South), Ministry of Agriculture, Institute of Edible Fungi, Shanghai Academy of Agricultural Sciences, Shanghai 201403, China

## Abstract

High-throughput technologies of functional genomics such as T-DNA insertional mutagenesis and microarray expression profiling have been employed to identify genes related to pathogenicity in* Magnaporthe oryzae*. However, validation of the functions of individual genes identified by these high-throughput approaches is laborious. In this study, we compared two published lists of genes putatively related to pathogenicity in* M. oryzae* identified by T-DNA insertional mutagenesis (comprising 1024 genes) and microarray expression profiling (comprising 236 genes), respectively, and then validated the functions of some overlapped genes between the two lists by knocking them out using the method of target gene replacement. Surprisingly, only 13 genes were overlapped between the two lists, and none of the four genes selected from the overlapped genes exhibited visible phenotypic changes on vegetative growth, asexual reproduction, and infection ability in their knockout mutants. Our results suggest that both of the lists might contain large proportions of unrelated genes to pathogenicity and therefore comparing the two gene lists is hardly helpful for the identification of genes that are more likely to be involved in pathogenicity as we initially expected.

## 1. Introduction

Rice blast caused by the fungal pathogen* Magnaporthe oryzae* is one of the most destructive diseases of rice threatening the sustainability of global food production.* M. oryzae* attacks various parts of rice including leaves, stems, nodes, and panicles [1, 2]. The infection of* M. oryzae* is a complex process [3, 4]. It begins from the attachment of a three-celled conidium to the rice leaf, followed by the germination of the conidium and the differentiation of the germ tube into a dome-shaped cell called appressorium. The appressorium matures and generates turgor by accumulating high concentrations of compatible solutes, which can directly penetrate the host cuticle, resulting in a disease lesion. After penetration, the bulbous branched infectious hyphae rapidly spread to adjacent cells and form conidiophores to release conidia into the environment so as to initiate new infection. In short, the infection process of* M. oryzae* consists of four stages: attachment, germination, differentiation, and penetration. Obviously, such a complex process must be controlled by a great number of genes.

To prevent and control rice blast efficiently, it is necessary to understand the mechanisms involved in this disease and find out genes related to the pathogenicity of* M. oryzae*. It is known that the interaction between rice and blast fungus complies with the gene-for-gene relationship. Therefore, avirulence genes play important roles in the pathogenicity of* M. oryzae*. However, many other genes involved in the infection process may also contribute to the pathogenicity. In recent years, a few gene manipulation approaches, such as homologous recombination [5–7], T-DNA insertional mutagenesis [8, 9], and RNA interference [10–12], have been employed to study the pathogenicity-related genes in* M. oryzae*. Some key genes for pathogenicity have been identified and cloned [4, 13–15].

As the complete genome sequence of* M. oryzae* has been available [16], large-scale and systematic identification of pathogenicity-related genes in the rice blast fungus becomes feasible. Jeon et al. [17] obtained a total of 21,070 mutants in* M. oryzae* through large-scale T-DNA insertional mutagenesis, from which over 1,000 genes putatively related to pathogenicity were identified using a high-throughput phenotype screening pipeline. Oh et al. [18] employed a whole genome oligo-DNA microarray of* M. oryzae* to analyze genome-wide gene expression changes during spore germination and appressorium formation. They detected hundreds of differentially expressed genes, which were thought to be related to appressorium formation (and therefore possibly to pathogenicity). These results will be helpful for deep research on the molecular mechanisms of pathogenicity in* M. oryzae*. However, the exact functions of these genes still need to be verified individually. This will be laborious work.

Since the gene list obtained by the T-DNA insertional mutagenesis [17] and that obtained by the microarray analysis [18] are both putatively related to pathogenicity, it would be interesting to see how many genes are overlapped between them and whether the overlapped genes are more likely to be pathogenicity-related. This would allow us to evaluate the reliability and usefulness of the gene lists. For this purpose, in this study, we examined the overlapping between these two gene lists and validated the functions of the overlapped genes by knocking them out using the method of target gene replacement.

## 2. Materials and Methods

### 2.1. Sources and Comparison of Gene Lists

Two gene lists of* M. oryzae* were used for the study. One was acquired from the Supplementary Tables  2 and  4 of Jeon et al. [17]. The list contained a total of 1024 putatively mutated genes identified from 547 T-DNA insertion mutant strains. ~90% of the mutants displayed changed phenotypes and/or weakened pathogenicity. There were two types of mutations: Type I is that a gene is disrupted by the T-DNA, which is inserted inside the gene, so that the gene's function is usually lost; Type II is that a gene remains intact in structure, but its function is possibly affected by the T-DNA, which is inserted beside it. Most (~88%) of the genes in this list belonged to Type I, only a small proportion (~12%) belonged to Type II. The other gene list was obtained from the Additional Data File 3 of Oh et al. [18]. The list contained 236 genes that were either upregulated (~2/3) or downregulated (~1/3) in response to the stimulation of hydrophobic surface during appressorium formation according to microarray analysis and therefore were thought to be possibly related to appressorium formation. For convenience, we shall call the two gene lists as MUG (mutated gene) list and DEG (differentially expressed gene) list, respectively. The overlapping between the two gene lists was examined.

### 2.2. Fungal Strains, DNA Extraction, and Southern Blot Analysis

The wild-type* M. oryzae* strain Guy11, which is pathogenetic to many rice varieties and can infect barley and the model grass species* Brachypodium distachyon* as well [4, 19], was used for the gene-knockout experiment. All* M. oryzae* strains (including Guy11 and its gene-knockout mutants) were cultured on CM medium. Genomic DNA of each strain was extracted using the CTAB method as described [20]. Restriction enzyme digestion and ligation were performed according to Sambrook and Russell [21]. The DIG high prime DNA labeling and Kit I (Roche, Germany) were used for Southern blot analysis.

### 2.3. Vector Construction and Transformation

Construction of target gene replacement vector and fungal transformation were performed as described [3, 22]. Putative gene deletion mutants were recovered and selected on a complete medium with 200 ug/mL hygromycin and further selected by three rounds of screening using PCR (1st round), Southern blot (2nd round), and RT-PCR (3rd round). Primers used for the gene replacement, PCR, and RT-PCR are listed in Table 1.

### 2.4. Phenotype Investigation and Pathogenicity Test

The growth characteristic, conidiation, conidial germination, appressorium formation, and conidial morphology of each* M. oryzae* strain were observed following the protocols as described [3, 22]. After a strain was cultured on CM medium for 10 days, conidia were collected and suspended in 0.2% (w/v) gelatin solution. After resuspension to a concentration of 1 × 10^5^ conidia/mL, the conidia were inoculated by spraying on the 14-day-old seedlings of rice (*Oryza sativa* L.) cultivar CO-39. The inoculated rice seedlings were placed in a chamber with a 12–24 h photophase under 25°C and then transferred to a greenhouse with 14 h of light and 10 h of dark for 7 days. Disease severity was rated according to the method of [23].

## 3. Results

### 3.1. Overlapping between the Two Gene Lists

Comparison between the MUG list and the DEG list indicated that the overlapping between them was very low (Table 2). Only 13 genes were overlapped between the two lists, accounting for 1.27% (13/1024) in the MUG list and 5.51% (13/236) in the DEG list, respectively. Among the 13 overlapped genes, eleven were upregulated and two were downregulated in response to the stimulation of hydrophobic surface during appressorium formation. Meanwhile, only two genes belonged to Type I, while most belonged to Type II. Noticeably, the proportion of Type I genes in the overlapped genes (2/13 ≈ 15.4%) was very close to that in the MUG list (~12%). In addition, eight (61.5%) genes were previously found to be possibly related to pathogenicity according to the phenotypes of their T-DNA insertion mutants. This proportion was smaller than that in the MUG list (~90%).

### 3.2. Phenotypic Effects of the Overlapped Genes

To evaluate the overlapped genes, we examined five of them, that is, MGG_00623, MGG_00745, MGG_00871, MGG_04068, and MGG_06704 (Table 2), by gene knockout using the method of target gene replacement. Considering that Type II genes were predominant in the MUG list and the function loss of a gene is usually ensured in Type I mutation but not in Type II mutation, the five genes we selected all belonged to Type II. In addition, these five genes were all upregulated in response to the stimulation of hydrophobic surface and were all previously proved to be possibly involved in pathogenicity as their T-DNA insertion mutants all exhibited visible phenotypic changes and pathogenicity reduction.

We failed to obtain null mutants of MGG_04068 gene although we performed four independent transformations and screened several hundreds of transformants. A possible reason is that the cell could not survive when MGG_04068 gene is knocked out. But the molecular function of MGG_04068 is not known. According to the annotation, it is a conserved hypothetical protein.

**Figure 1:**
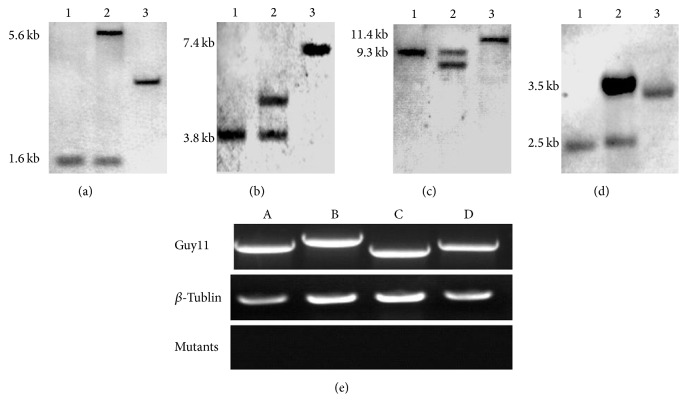
Southern blot and RT-PCR analysis of the transformants. (a) MGG_00623. DNA samples were digested with* Apa*I, probed with a 1.2 kb upstream flanking sequence fragment of the gene replacement vector. The 5.6 kb band was detected in mutant, whereas the 1.6 kb band was detected in wild-type Guy11; (b) MGG_00871. DNA samples were digested with* Kpn*I, probed with a 1.0 kb upstream flanking sequence fragment of the gene replacement vector. The 7.4 kb band was detected in mutant, whereas the 3.8 kb band was detected in wild-type Guy11; (c) MGG_06704. DNA samples were digested with* Sac*I, probed with a 1.3 kb downstream flanking sequence fragment of the gene replacement vector. The 11.4 kb band was detected in mutant, whereas the 9.3 kb band was detected in wild-type Guy11; (d) MGG_00745. DNA samples were digested with* EcoR*V, probed with a 1.0 kb upstream flanking sequence fragment of the gene replacement vector. The 3.5 kb band was detected in mutant, whereas the 2.5 kb band was detected in wild-type Guy11; (a–d) Lane of 1: Guy11; 2: transformant with selection marker but not single copy; 3: mutant with targeted gene had been replaced. (e) Lane of A: ΔMGG_00623; B: ΔMGG_00871; C: ΔMGG_06704; D: ΔMGG_00745.

**Figure 2:**
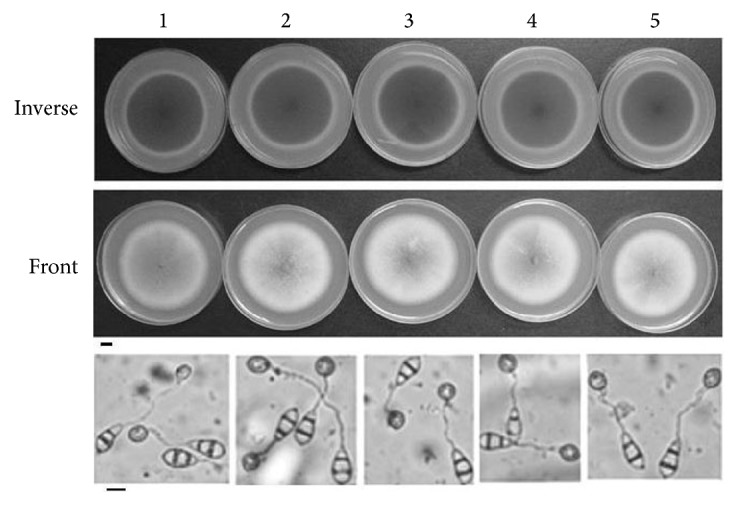
Pigmentation and Morphology of conidia and appressorium of* M. oryzae* strains. The wild-type Guy11 and mutants were grown on CM medium for 11 days, and colonies were photographed; we had not found change on pigmentation. Bar: 1 cm; the pictures of appressorium were photographed after the conidia induced 6 h on film. Bar: 10 *μ*m. Number of 1: Guy11; 2: ΔMGG_00623; 3: ΔMGG_00871; 4: ΔMGG_06704; 5: ΔMGG_00745.

**Figure 3:**
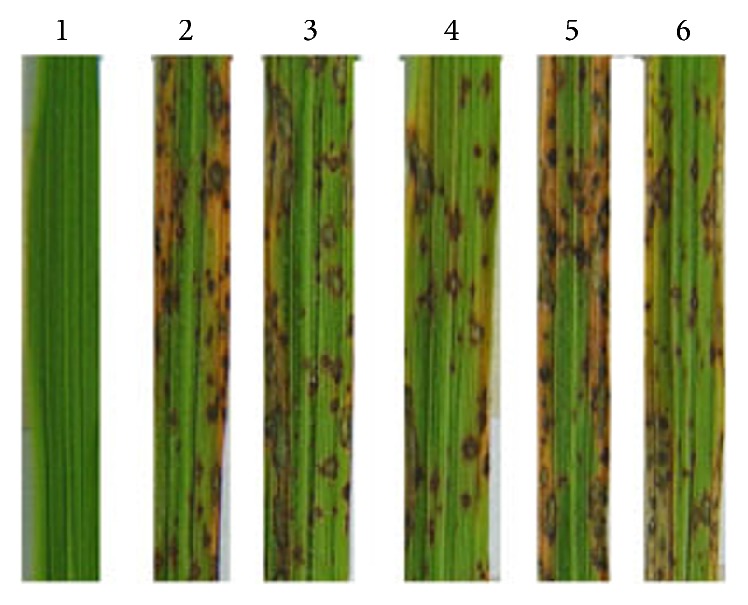
Leaves from CO-39 were spray inoculated individually with conidia. Photographed images 7 days after rice seedlings were inoculated with conidia (1 × 10^5^ conidia/mL) from the wild-type Guy11 and mutants; Number of 1: 0.2% gelatin (control); 2: Guy11; 3: ΔMGG_00623; 4: ΔMGG_00871; 5: ΔMGG_06704; 6: ΔMGG_00745.

The other four genes were all successfully knocked out, confirmed by Southern blot and RT-PCR (Figure 1). However, the null mutants of the four genes did not show significant phenotypic changes on growth characteristic (GC; Table 3), pigmentation (PG; Figure 2), conidiation (Table 4), conidial germination (Table 4), appressorium formation (Table 4), and conidia and appressorium morphology (Figure 2), suggesting that these genes have little effect on fungal development. Moreover, after inoculation, these mutants all resulted in typical spindle-like and gray-center lesions in rice seedling leaves with similar disease score to that caused by wild-type Guy11 (Figure 3). The result indicates that these four genes are not necessary for pathogenicity in* M. oryzae*.

## 4. Discussion

T-DNA insertional mutagenesis and microarray expression profiling are two powerful technologies for functional genomics research. Although the two technologies analyze gene functions from different aspects, it can be expected that the lists of genes identified by them for the same traits would tend to be overlapped or positively correlated. Based on this consideration, in this study, we tried to screen genes that would be more likely to be related to pathogenicity in* M. oryzae* by comparing the MUG and DEG lists. However, we were surprised to find that the overlapping between the two gene lists was terribly low (Table 2).

Based on the genome sequence draft of* M. oryzae*, it was estimated that there are totally 11109 genes in* M. oryzae* [16]. According to this estimate, we can find that the number of overlapped genes between the MUG and DEG lists is expected to be 1024 × 236/11109 ≈ 22 provided the two gene lists are independent random samples from the whole set of* M. oryzae* genes. Obviously, if the two gene lists are positively correlated, the number of overlapped genes will be much greater than this value. But the actual number we found was 13, even smaller than that. This result suggests that the two gene lists are not positively correlated as expected.

In the subsequent experiment for validating gene function, we successfully knocked out four Type II overlapped genes. The T-DNA insertion mutants of these four genes all displayed phenotypic changes and pathogenicity reduction (Table 1; [17]), but none of the knockout mutants of these four genes we obtained exhibited any phenotypic changes and pathogenicity reduction as expected. Hence, according to our result, these four genes are not related to pathogenicity. Suppose the proportion of pathogenicity-unrelated genes among the Type II overlapped genes is *p*, then the probability that all the four genes examined are not related to pathogenicity will be *p*^4^. Following the principle that an event of small probability is unlikely to occur in a single experiment, we may require *p*^4^ > 0.05, which means *p* > 0.473, or half of the Type II overlapped genes would be pathogenicity-unrelated. As this is a conservative estimate, the real proportion of pathogenicity-unrelated genes might be higher.

Taken together, our study indicates that the MUG list and the DEG list only have a small overlapping, and at least half of the overlapped genes are not related to pathogenicity. These two results are consistent, both of which suggest that the two gene lists are basically independent and at least one of the two gene lists is close to a random sample in regard to pathogenicity. We suspect that the DEG list is more likely to be the case. It is possible that in the DEG list most of the genes responding to the stimulation of hydrophobic surface are not involved in pathogenicity. In our experiment, we also knocked out another four genes in the DEG list but not among the overlapped genes, and these four genes also did not show any phenotypic changes and pathogenicity reduction (results not presented). Hence, we totally examined eight genes in the DEG list by gene knockout and these genes all showed negative results. This suggests that there must be a large proportion of genes that are not related to pathogenicity in the DEG list, making the DEG list behave as a random sample approximately.

The MUG lists may also contain a large proportion of pathogenicity-unrelated genes. We have seen that the MUG list mainly consists of Type II genes (~88%). Generally speaking, Type II genes are resulted from the T-DNA insertion between two adjacent genes. In other words, each outside-gene T-DNA insertion can result in two Type II genes. Hence, in general, in each mutant containing an outside-gene T-DNA insertion, only one of Type II genes would possibly be the cause of the mutant phenotypes, while the other one would be unrelated. In addition, sometimes there can be multiple copies of T-DNA insertion in a mutant. In this case, the number of unrelated genes among the Type II genes will be increased. Therefore, we can expect that at least half of the Type II genes in the MUG list are not related to pathogenicity.

## 5. Conclusion

Based on the above discussion, we think that the large proportions of pathogenicity-unrelated genes in the MUG and DEG lists (especially the latter) must be the major reason for the low overlapping or little correlation between the two gene lists. Our results suggest that comparing the two types of gene lists does not facilitate the identification of genes that are more likely to be involved in pathogenicity as we initially expected. Hence, how to efficiently validate the functions of genes identified by T-DNA insertional mutagenesis and microarray expression profiling is still an arduous task. Analysis of double or multiple mutants would be an effective approach for determining the functions of genes discovered by T-DNA random insertion [24].

## Figures and Tables

**Table 1:** The primers used for genes replacement and PCR in this study.

Primers name	Sequence of PCR primers	Application of primers
P1UFMGG-00623	ctcgagATTCGGGTCCTTCGTTAT	For genes replacement
P1URMGG-00623	gtcgacCCTCCCTCTGTTGTCTTGT	For genes replacement
P1DFMGG-00623	actagtGACCGTGATCGACCTTCC	For genes replacement
P1DRMGG-00623	gagctcATGCCCTCTTTGACTTGG	For genes replacement
P2UFMGG_00871	ggtacc TCGAGGGTTATCAAGCAA	For genes replacement
P2URMGG_00871	gtcgacAAATAGAAGCCGCCAGAC	For genes replacement
P2DFMGG_00871	gaattcGATGACGAGTTGCGATGT	For genes replacement
P2DRMGG_00871	tctagaGGGACCTGCTCTGTATCA	For genes replacement
P3UFMGG_06704	gggcccCCGTCATCACCTAACCAA	For genes replacement
P3URMGG_06704	ctcgagGAACAGCGTCGTCTCCAT	For genes replacement
P3DFMGG_06704	actagtGACCGTGATCGACCTTCC	For genes replacement
P3DRMGG_06704	gagctcATGCCCTCTTTGACTTGG	For genes replacement
P4UFMGG_00745	ctcgagGCGGGTCAAAGAGTGTATT	For genes replacement
P4URMGG_00745	gagctcGTCGTTGGGTATTGGGTC	For genes replacement
P4DFMGG_00745	gaattcCACTTCTTTCCCTGGTCG	For genes replacement
P4DRMGG_00745	gagctcTCCTCTGGAGCTTTCCTC	For genes replacement
P5UFMGG_04068	gggcccGGGGCAAGGTTCTCAAAG	For genes replacement
P5URMGG_04068	gtcgacAAGCGAGGTGGCAGGTAG	For genes replacement
P5DFMGG_04068	aagcttAGGTCGTAGACATACTGAGGT	For genes replacement
P5DRMGG_04068	gaattcAAGGCTGTAGATGGCTGA	For genes replacement
CP1FMGG-00623	ACTTGATGGCTAACCACTACTT	For PCR screening
CP1RMGG-00623	CCAATATGTCCGAGACGAT	For PCR screening
CP2FMGG_00871	CCCATTGATACTGCGGTTAG	For PCR screening
CP2RMGG_00871	TTGATCGTGCCGTCCTCT	For PCR screening
CP3FMGG_06704	CATCGTGGACATCTTGGAG	For PCR screening
CP3RMGG_06704	CGAAACTTCTGGTGGTGAT	For PCR screening
CP4FMGG_00745	CTCCGTTGCGTCGTCTGT	For PCR screening
CP4RMGG_00745	TCTGGTCCGTCTTGCTGTT	For PCR screening
CP5FMGG_04068	ATCACAACCCTCCGAACCA	For PCR screening
CP5FMGG_04068	GCAAACCTGTCCTCGTAGTCC	For PCR screening
R-P1FMGG-00623	CGCATCCCAAGCCTGAAT	For RT-PCR screening
R-P1RMGG-00623	AGAACGGCGGGTGACAAG	For RT-PCR screening
R-P2FMGG_00871	AAGGGTCCGACGAGCAAA	For RT-PCR screening
R-P2RMGG_00871	CCTCCAACTCCACGGGTAT	For RT-PCR screening
R-P3FMGG_06704	GGAGTGGGAGGACAATGAA	For RT-PCR screening
R-P3RMGG_06704	GTCGCAATGGCAAGAACA	For RT-PCR screening
R-P4FMGG_00745	GGACCCAATACCCAACGAC	For RT-PCR screening
R-P4RMGG_00745	ACGGCTCATACGGCATAAA	For RT-PCR screening

**Table 2:** Information of 13 overlapping genes (“+” indicate change).

Gene name	Regulation	Insert location	Mutant ID	GR^a^	PG^b^	CN^c^	GM^d^	AP^e^	CM^f^	PT^g^
MGG_00450	Up	MGG_00450	0137A2, 0128D5	+	+	+		+		+
MGG_00623	Up	MGG_00623-MGG_00624	0035C2							+
MGG_00745	Up	MGG_00744-MGG_00745	0059A3					+		+
MGG_00871	Up	MGG_00870-MGG_00871	0430D2					+		+
MGG_00994	Up	MGG_00994-MGG_11455	0673D3, 0690A4			+				+
MGG_01778	Up	MGG_01778-MGG_01779	0008C4							
MGG_02763	Up	MGG_02763-MGG_12596	0156D5							
MGG_02817	Down	MGG_02817	0010A5							
MGG_04068	Up	MGG_04068-MGG_04069	0257C4			+			+	
MGG_06704	Up	MGG_06704-MGG_06705	0416A3, 0420C2					+	+	+
MGG_09096	Up	MGG_09096-MGG_09095	0007B2							
MGG_09200	Down	MGG_09200-MGG_11770	0059B3					+		+
MGG_09942	Up	MGG_09942-MGG_09941	0236B3							

^a^GR: growth rate; ^b^PG: pigmentation; ^c^CN: conidiation; ^d^GM: conidial germination; ^e^AP: appressorium formation; ^f^CM: conidial morphology; ^g^PT: pathogenicity.

**Table 3:** Growth characteristic of *M. oryzae* strains^A^.

*M. oryzae*	Growth days after				
	3	5	7	9	11
Guy11	2.09 ± 0.18^a^	3.43 ± 0.23^a^	5.20 ± 0.00^a^	6.61 ± 0.01^a^	8.02 ± 0.00^a^
ΔMGG_00623	2.08 ± 0.05^a^	3.46 ± 0.05^a^	5.1 ± 0.00^a^	6.55 ± 0.00^a^	7.80 ± 0.20^a^
ΔMGG_00871	2.03 ± 0.17^a^	3.46 ± 0.15^a^	5.00 ± 0.19^a^	6.41 ± 0.05^a^	7.90 ± 0.00^a^
ΔMGG_06704	2.08 ± 0.14^a^	3.45 ± 0.14^a^	5.00 ± 0.17^a^	6.39 ± 0.20^a^	7.87 ± 0.11^a^
ΔMGG_00745	2.18 ± 0.05^a^	3.62 ± 0.07^a^	5.05 ± 0.22^a^	6.53 ± 0.11^a^	7.93 ± 0.15^a^

^A^The diameter of the wild-type Guy11 and mutants was measured after inoculation in CM plates for 3, 5, 7, 9, and 11 days. The targeted genes replacement had no distinguishable effect on growth rate. The letters “a” in each column are not significantly different, as estimated by Duncan's test (*p* ≤ 0.05).

**Table 4:** Conidiation, conidial germination, and appressorium formation of the wild Guy11 and mutants.

*M. oryzae*	Conidiation (×10^4^ cm^−2^)^A^	Conidial germination (%)^B^	Appressorium formation (%)^B^
		4 h	12 h
Guy11	117.85 ± 27^a^	91.23 ± 2.79^a^	90.62 ± 3.71^a^
ΔMGG_00623	120.71 ± 26^a^	92.93 ± 2.20^a^	90.11 ± 1.62^a^
ΔMGG_00871	105.71 ± 41^a^	90.70 ± 3.84^a^	90.80 ± 4.17^a^
ΔMGG_06704	114.28 ± 48^a^	92.74 ± 2.26^a^	91.08 ± 4.47^a^
ΔMGG_00745	117.85 ± 55^a^	92.10 ± 2.64^a^	92.09 ± 3.08^a^

^A^After incubation for 10 days on CM plates, conidia were collected using three 1 cm diameter discs of mycelium in water and counted with a haemacytometer under a microscope.

^B^Conidial germination and appressorium formation were recorded after different time incubation of the conidial suspension on the hydrophobic surface of film as described previously. The letters “a” in each column are not significantly different, as estimated by Duncan's test (*p* ≤ 0.05).
